# Cultural diversity: blind spot in medical curriculum documents, a document analysis

**DOI:** 10.1186/1472-6920-14-176

**Published:** 2014-08-22

**Authors:** Emma Paternotte, Joanne PI Fokkema, Karsten A van Loon, Sandra van Dulmen, Fedde Scheele

**Affiliations:** 1 Department of Medical Education, Sint Lucas Andreas Hospital, Jan Tooropstraat 164, P.O. Box 9243, 1061 AE Amsterdam, The Netherlands; 2 Department of Primary and Community Care, Radboud University Medical Centre, Nijmegen, The Netherlands; 3 NIVEL (Netherlands Institute for health services research), Utrecht, The Netherlands; 4 Department of Health Sciences, Buskerud and Vestfold University College, Drammen, Norway; 5 Department of Medical Education, VU University Medical Centre, Amsterdam, The Netherlands

## Abstract

**Background:**

Cultural diversity among patients presents specific challenges to physicians. Therefore, cultural diversity training is needed in medical education. In cases where strategic curriculum documents form the basis of medical training it is expected that the topic of cultural diversity is included in these documents, especially if these have been recently updated. The aim of this study was to assess the current formal status of cultural diversity training in the Netherlands, which is a multi-ethnic country with recently updated medical curriculum documents.

**Methods:**

In February and March 2013, a document analysis was performed of strategic curriculum documents for undergraduate and postgraduate medical education in the Netherlands. All text phrases that referred to cultural diversity were extracted from these documents. Subsequently, these phrases were sorted into objectives, training methods or evaluation tools to assess how they contributed to adequate curriculum design.

**Results:**

Of a total of 52 documents, 33 documents contained phrases with information about cultural diversity training. Cultural diversity aspects were more prominently described in the curriculum documents for undergraduate education than in those for postgraduate education. The most specific information about cultural diversity was found in the blueprint for undergraduate medical education. In the postgraduate curriculum documents, attention to cultural diversity differed among specialties and was mainly superficial.

**Conclusions:**

Cultural diversity is an underrepresented topic in the Dutch documents that form the basis for actual medical training, although the documents have been updated recently. Attention to the topic is thus unwarranted. This situation does not fit the demand of a multi-ethnic society for doctors with cultural diversity competences. Multi-ethnic countries should be critical on the content of the bases for their medical educational curricula.

## Background

In multi-ethnic societies, providing effective healthcare is challenged by various aspects of cultural diversity, such as epidemiological health differences between populations, communication barriers and differences in religion, socio-economic status and ethnic background [1]. During the past decade, various studies have demonstrated that the increase in cultural diversity in many patient populations presents specific challenges to healthcare providers [2,3]. For instance, ethnic minority patients in developed countries, visit the physician more often [4], have longer visits [3] and are less satisfied with the physician-patient contact [5-7]. In addition, language barriers have been shown to diminish healthcare outcomes [6], and some ethnic groups have prolonged hospital stays and more unplanned readmissions [3].

To provide good quality of care, physicians need to be able to acknowledge, recognize and deal with these challenges. Therefore, cultural diversity should be addressed in medical training [8-12]. In multi-ethnic countries, cultural diversity is considered an essential topic in society [8,11,13], which needs to get attention in medical training to prepare students for their work as physicians [13].

To ensure adequate attention to cultural diversity, cultural diversity training should be anchored in strategic curriculum documents for medical education in multi-ethnic countries. Ten to fifteen years ago, overviews of curricula of medical education in the United States of America (USA), Canada, the United Kingdom (UK) and the Netherlands showed that cultural diversity training was scarcely addressed and that students’ preparation for cultural issues was inadequate [1,9,14]. Since then, however, cultural diversity in medical education has been identified as a point of interest in the Netherlands, as in many other Western countries [2,9,14,15]. Also, in recent years, there have been several occasions for revising the content of programs and for including cultural diversity in the curriculum documents. For example, in the Netherlands, the training programs for undergraduates were recently inspected and the curriculum documents for postgraduates were recently revised [16].

Since cultural diversity training is considered essential for physicians [8,11,12], it is important to know if cultural diversity has gained more attention in curriculum documents over the last years. Insight into the current status of cultural diversity in strategic curriculum documents is required to assess whether the conditions for effective curriculum development in this area are met.

The aim of this study was to assess the formal status of cultural diversity training in a multi ethnic country. In particular, we studied the formal status of cultural diversity training in the Netherlands, a country with 17 million inhabitants, 3.5 million (20%) of whom are members of ethnic minority groups [17]. Although not composed of various ethnic groups since its foundation, the Netherlands has been a diverse country for a long time. Migration to the Netherlands started in the 17^th^ century and after that the Netherlands experienced a growing migration since the 1960’s because of its growing prosperity and the following migration for work [18]. This ethnic diversity currently ranges from a Moroccan population to Turkish, Surinam and Western migrants [17]. We conducted a document analysis focusing on the current attention to the topic of cultural diversity training in strategic curriculum documents that form the basis of actual training. The question that guided our research was: To what extent and how is attention to cultural diversity ensured in the strategic curriculum documents that guide medical education in the Netherlands?

## Methods

### Setting

**Figure 1:**
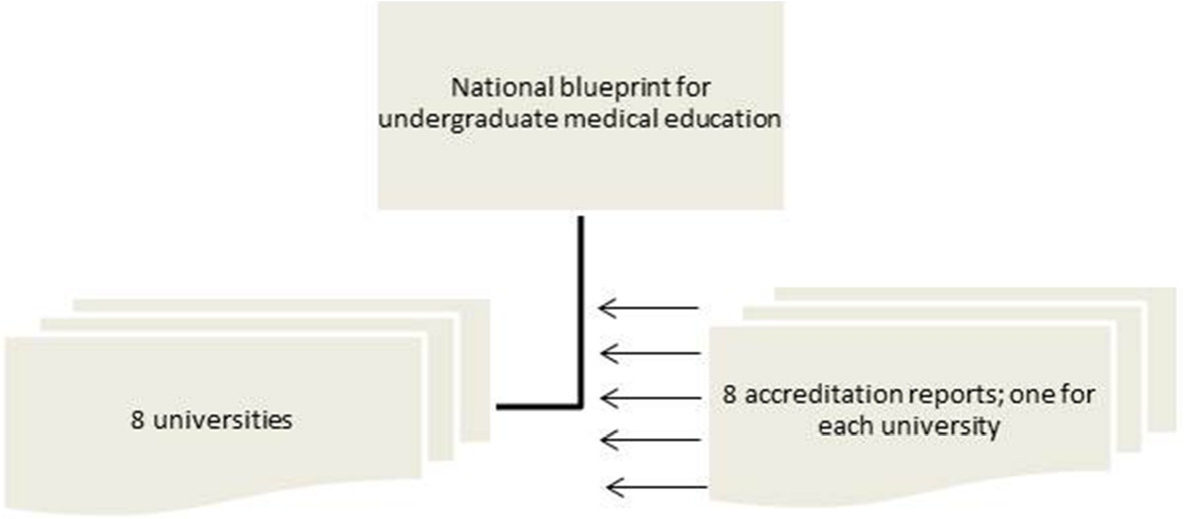
The used curriculum documents for undergraduate medical education in the Netherlands.

**Figure 2:**
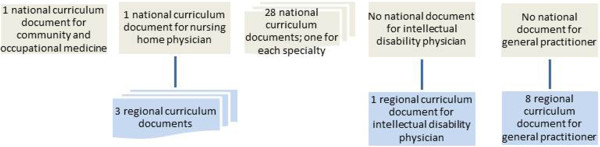
The used curriculum documents for postgraduate medical education in the Netherlands.

We conducted this study on curriculum documents of the Netherlands, as a case of a country with a culturally diverse patient population and recently revised curriculum documents for medical education. Medical education in the Netherlands consists of undergraduate and postgraduate medical education (UGME and PGME). Undergraduate education is provided by all 8 universities in the country, which all have a university teaching hospital. Postgraduate specialty education is executed in eight regions of which each contains one of the university teaching hospitals and several affiliated general teaching hospitals. Actual training is executed in the hospitals, which is referred to as "locally". Both UGME and PGME are directed by national and regional curriculum documents. These are all policy documents and serve as guidelines for the taught curriculum. The documents describe the requirements and goals which should be fulfilled at the end of the training, using the roles described by the Canadian Medical Education Directives for Specialists (CanMEDS) [19]. The national documents are developed by project groups of concerned stakeholders which are coordinated by the national organization Royal Dutch Medical Association [20]. This organization insists on the quality of medical profession and healthcare. For undergraduate medical education (see Figure 1) the national document is the blueprint. The blueprint was introduced in 1994 and rewritten in 2009 to define student’s learning outcomes. For postgraduate medical education (see Figure 2) national curriculum documents are concentrated to specific specialty training. Some specialty training does not have a national curriculum document, because some training is only given in one region. In these cases, we used regional documents.

For undergraduate medical education only describing the blueprint could be too superficial, because of its intended nature to only function as a guideline. Therefore we decided to include the accreditation reports of the 8 universities in the Netherlands as well. This accreditation is done for every university separately by a commission of external experts, which checks if the rules of the blueprint are followed. This is done every four year or more frequently if the commission decides so [21]. These documents could be seen as regional documents. We included these documents to gain a deeper insight into the point of interest and improvements of every university.

### Design

To describe the formal status of cultural diversity training, we performed a document analysis of the UGME and PGME curriculum documents. As a basis, we used the educational framework of the Accreditation Council for Graduate Medical Education (ACGME) [19], which focuses on three domains: objectives, methods and evaluation. Objectives are the competences (knowledge, skills and professional behavior) that have to be acquired by the trainees. The training methods explain how these competences should be attained, and evaluation indicates how achievement of the objectives should be examined.

The three domains are generally presented in this systematic order [19], and their inclusion can be considered a requirement for adequate curriculum design [22]. For example, a competence described in the curriculum document of the postgraduate training for gynecologist is *‘the support of a physiological delivery’*. The objective for this competence is that residents demonstrate to support an uncomplicated delivery without supervision. The training method used is the exercise on the phantom, and the final evaluation consists of practical exam on the phantom.

### Procedure

The strategic curriculum documents were retrieved through internet searches in February and March 2013. Documents that were not available on the internet were requested from program directors by email [20,21,23]. On the advice of program directors of the undergraduate medical education, we also retrieved the national blueprint (a national policy document for medical undergraduate education) [24] and the accreditation reports that Quality Assurance Netherlands Universities (QANU) made of the 8 universities that provide a medical curriculum. The accreditation reports of medical education contained evaluations of all the bachelor and master programs [21]. One university’s undergraduate accreditation report was not available at the moment of analyzing the data. Instead, this university provided a summary of the cultural diversity objectives mentioned in their accreditation report. For the purpose of this study, cultural diversity was defined as a difference in ethnic background between a physician and his or her patient [25].

### Analysis

The first author (EP) systematically read the strategic curriculum documents and extracted all phrases about cultural diversity. Text phrases of the documents which mentioned cultural diversity (i.e. diversity, cultural, intercultural, ethnicity) were sorted into the three domains of the ACGME framework, objective, method and evaluation [19]. To interpret the meaning of the extracted phrases about cultural diversity, this was an iterative process [26]. Doubts concerning the inclusion of text phrases and their position in the framework were discussed with co-authors JF and KL. There was disagreement about three phrases, which all concerned mini-CEX. After discussion with all members of the research team whether these should be considered methods or evaluation tools three phrases were changed from evaluation tools into methods.

## Results

In total, 52 documents were analyzed. For undergraduate education, we analyzed one national document, 7 regional curriculum accreditation reports and one summary. For postgraduate education, we analyzed 31 national curriculum documents and 12 regional curriculum documents. Text phrases about cultural diversity were found in 33 of these documents. In 6 of these, a specific text referred to cultural diversity. In 2 out of 52 documents, cultural diversity was referred to in all three domains, objective, training method and evaluation, and in the appropriate sequence. A summary of the findings is presented in Table 1.

**Table 1:** Summary of number of documents with text phrases regarding cultural diversity training in medical education

**Training**	Total documents (nat/reg *)	**In *n *** **documents phrases of cultural diversity**			
		**Objectives (O)**	**Methods (M)**	**Evaluation (E)**	**Combination (O + M + E) †**
Undergraduate training, national	1 (nat)	1	0	1	0
Undergraduate training, accreditation	8 (reg)	0	0	0	0
Graduate training: community and occupational medicine	2 (nat)	1	0	0	0
Graduate training: nursing home physician	4 (1 nat/3 reg)	4	0	0	0
Graduate training: general practitioner	8 (reg)	1	1	0	0
Graduate training: intellectual disability physician	1 (reg)	1	0	0	0
Graduate training: clinical residency training	28 (nat)	17	5	2	2

* national/regional

† In *n* documents a combination of objective, methods and evaluation was mentioned in one sequence.

### Cultural diversity in curriculum documents for undergraduate education

The Dutch national blueprint for undergraduate medical education was found to contain several objectives regarding cultural diversity. These objectives are formulated within the CanMEDS roles of Communicator, Medical expert and Health advocate. For example, in the description of the role Communicator, cultural diversity is specified as *"The student adequately handles diverse groups of patients, such as children, elderly, men, women and patients from different cultural backgrounds"*.

Attention to training methods was not found in the blueprint. In contained the recommendation that requirements, which should be fulfilled at the end of the programs, should be realistic and trainable, but no description is given of training methods. Regarding evaluation, it contained an appendix with a skills list that takes cultural aspects into account (evaluation). For example, *"Does the student indicate the influence of ethnic diversity on the healthcare process?"*

Compared to the national blueprint, fewer references were found in the accreditation reports. Of 7 regional accreditation reports and one summary of an accreditation report on undergraduate training, 3 did not mention cultural diversity, whereas 5 did address themes concerning cultural diversity. The cultural diversity themes described in these 5 documents were ‘learning medical ethics and diversity management’ , ‘acquiring cultural competence’ , ‘offering obligatory education about cultural diversity’ and ‘global health training’. Three of these 5 documents contained a small section that defined the term ‘cultural competence’.

### Cultural diversity in curriculum documents for postgraduate education

#### General practitioner

Two out of 8 regional strategic curriculum documents for the specialty ‘general practitioner’ contained a description of cultural diversity themes. One of these described the *"changing population’s demands on care"*, but this objective was not followed by a description of methods or evaluation. The other document contained a training method description referring to an elective course on multicultural care, which was not followed by an evaluation nor preceded by objectives. The other 6 documents contained no reference to cultural diversity training.

#### Community and occupational medicine

The national curriculum document on the specialty of community and occupational medicine is split into two documents, a manual and a curriculum. One of these, the manual, cultural diversity was addressed. This description was placed among the objectives, as part of the role of Communicator. It was not followed by a description of a training method or an evaluation.

#### Nursing home physician

There are 4 national and regional strategic curriculum documents for the specialty ‘nursing home physician’ , all of which offered a description of the role of Communicator in the context of a different cultural background of the patient (objective). These documents contained no phrases concerning methods or evaluation of cultural diversity training.

#### Intellectual disability physician

The regional strategic curriculum document for the specialty ‘disability medicine’ mentioned one CanMEDS role in the context of cultural diversity training; the role of Health advocate. This was followed by a brief reference to training method, *"The student integrates development and implementation of general medical insights with population-specific characteristics"*, without any reference to evaluation.

#### Clinical residency trainings

Ten out of 28 curriculum documents for clinical residency training did not mention cultural diversity. Cultural diversity was mentioned in 18 of the 28 documents on clinical residency training. In 17 of these 18 documents, cultural diversity objectives were described. These were formulated within various roles: Collaborator, Professional, Medical expert, Communicator, Health advocate or Reflector, which is a newly coined role. In 4 documents the objective was followed by a method, and in 2 of these, psychiatry and emergency medicine, the objective and method were followed by an evaluation. The training methods were the Mini-Clinical Evaluation Exercise (Mini-CEX) and *"The student should see a diverse patient population".* The evaluation consisted of observing the student in the context of cultural diversity, and of considering: *"Does the student recognize culture-specific presentations?"*

One of the 18 documents only described a method *("The student should see a diverse patient population"*), which was not preceded by an objective nor followed by an evaluation. In 2 of the 18 documents, cultural competence was generally mentioned as necessary for a physician.

## Discussion

This document analysis provided an impression of the formal status of cultural diversity in medical education in a multi ethnic country. We discovered that only half of all strategic curriculum documents contained references to cultural diversity training. Cultural diversity aspects were more prominently described in the curriculum documents for UGME than in those for PGME. The most specific information about cultural diversity was found in the blueprint for UGME. In the postgraduate curriculum documents, attention to cultural diversity differed among specialties and was mainly superficial. We found a remarkable absence of a systematic sequence of training objectives, training methods and evaluation, while this is regarded as important for adequate curriculum design [19].

Our finding of the amount of attention to cultural diversity resemble the results of the studies of Dogra et al. and Lu et al., who also described a remarkable absence of clearly described content for cultural diversity training in other countries [27,28]. They suggested that explanations for the missing content could be the challenges for the construction of a curriculum in ethnically diverse countries [14,15,27] and lack of universal core contents and standards. Another reason might be competition in an overloaded curriculum [28]. Furthermore, there is no clear consensus about the content that ought to be included in a cultural competence curriculum for physicians [29]. Still, there are also many initiatives worldwide to raise awareness for cultural competence in medical education for healthcare workers, national [30-32] and local [33]. In the USA for example, a strategy to incorporate cultural competence into training programs was developed [30]. Other examples are the UK [34] and Canada [35] where cultural diversity training for doctors is initiated.

One of the strengths of our study was that it was performed in a country with recently modernized curricula, which could be assumed to be updated according to recent insights into the requirements of a multi-cultural patient population. Our findings can serve as a basis for further research on the actual frequency and quality of cultural diversity training in medical education in newly ethnic diverse countries. A limitation of the study is that documents do not need to reflect the actual frequency and quality of cultural diversity training in educational practice, since the documents y often contain abstract formulations. On the other hand, the fact that cultural diversity is mentioned in the curriculum documents does not ensure that attention is given to this subject in actual practice.

## Conclusion

In conclusion, the importance of cultural diversity training has become apparent in Dutch undergraduate curriculum documents over the past ten years, although the vague and abstract terms used in these documents still need to be translated into practical guidelines for curriculum design. In postgraduate curriculum documents, there is little to no evidence that recent innovations in the Dutch medical curriculum have included improved attention to cultural diversity training, even though it is widely acknowledged to be necessary for all physicians who wish to deliver the highest quality of care. Thus, despite public recognition that cultural diversity competences are important for doctors in a multi-ethnic society, this recognition alone has not been sufficient to ensure adequate attention to cultural diversity training in medical curricula of newly diverse countries. This study could help to raise awareness among curriculum designers and could give leads for the development of a cultural competent curriculum.

## Competing interests

The authors declare that they have no financial competing interests and no non-financial competing interests. The authors alone are responsible for the content and writing of the paper.

## Authors’ contributions

EP carried out the searches for the documents, screened the documents to extract the appropriate text phrases and analyzed the data. JF checked and analyzed the extracted data of the documents, and helped to draft the manuscript. KL checked and analyzed the data of the documents. SD conceived of the study, and participated in its design and helped to draft the manuscript. FS helped with searching the documents, participated in the design and helped to direct the discussions. All authors read and approved the final manuscript.

## Pre-publication history

The pre-publication history for this paper can be accessed here:

http://www.biomedcentral.com/1472-6920/14/176/prepub
